# The Expression of Microvesicles in Leukemia: Prognostic Approaches

**DOI:** 10.22074/cellj.2019.5847

**Published:** 2019-02-25

**Authors:** Ali Ehsanpour, Najmaldin Saki, Marziye Bagheri, Masumeh Maleki Behzad, Saeid Abroun

**Affiliations:** 1 Thalassemia and Hemoglobinopathy Research Center, Research Institute of Health, Ahvaz Jundishapur University of Medical Sciences, Ahvaz, Iran; 2 Department of Hematology, Faculty of Medical Sciences, Tarbiat Modares University, Tehran, Iran

**Keywords:** CD Markers, Leukemia, microRNAs, Microvesicles, Prognosis

## Abstract

Microvesicles (MVs) are the smallest subclass of the extracellular vesicles (EVs) spontaneously secreted by the external budding from the cell membranes in physiologic and pathologic conditions. The MVs derived from leukemic cells (LCs) can be detected by the expression of specific cluster of differentiation (CD) markers indicating their cellular origin while they can transfer different agents such as microRNAs, cytokines, and chemokines. The secretion of these agents from MVs can affect the vital processes of LCs such as cell cycle, proliferation, differentiation, and apoptosis. According to the effects of MVs components on the vital processes of LCs, it has been postulated that a change in the expression of MVs might be involved in the progression and prognosis of leukemia. However, further studies are needed to confirm the association between the presence of MVs and their components with the prognosis of leukemia. It seems that the identification of the prognostic values and the application of them for the detection of MVs in leukemia can provide new therapeutic targets for monitoring the status of patients with leukemia.

## Introduction

A cell can secrete the different types of vesicles, the extracellular vesicles (EVs), into the extracellular environment with a range from a few nanometers to several microns in size (1). According to either the size or source, EVs are divided into three subclasses: apoptotic bodies, microvesicles (MVs), and exosomes (2). MVs are the smallest subclass of vesicles, which differ in size from between 100 nm and 1000 nm, and they are secreted by the external budding from the membrane of the normal cells such as platelets, endothelial cells, and leukocytes (2-4). It has been shown that these secretory factors can also be released under pathological conditions such as cancers, inflammation, coronary heart disease, diabetes, pre-eclampsia, and hematological malignancies (3, 4). Despite their small size, MVs can contain several biological agents such as growth factors, enzymes, adhesion molecules, and nucleic acids including microRNAs (miRNAs) (5, 6). The production rate of MVs and the cellular linage markers for their membranes are different depending on the cell origin (7).

MVs can affect the cell fate via direct binding to receptors of the target cell there by secreting their components into the extracellular medium, as well as endocytosis (3, 8, 9). However, MVs do not transfer their components into target cells in a random manner, but their secretion is regulated by several small GTPases such as ADP-ribosylation factors 1 and 6 (ARF1 and ARF6), rhodopsin A (Rho A), Rac family small GTPase 1 (Rac1), and Rab (6, 9). Indeed, these GTPases can indirectly regulate the MVs secretion pathways (6). miR-containing MVs can lead to genetic changes in the target cell due to their effect on the expression regulation of specific genes (10-12). 

These genetic changes can indirectly affect the vital processes such as differentiation, proliferation, and apoptosis (12, 13). In addition, MVs can also participate in biological processes such as thrombosis because of the transportation of the other components such as tissue factor (TF), cytokines, and chemokines receptors (4). Therefore, the elevated levels of TF- containing MVs can be associated with reduced survival in patients.

Leukemias are a group of hematological malignancies namely lymphocytic and myelocytic leukemia which is further divided into the acute and chronic types depending on the origin of the cell types and clinical manifestations, respectively. The hallmark of these malignancies is an increase in leukemic cells (LCs) in bone marrow (BM) and their release into the peripheral blood (PB) (14). It has been shown in these malignancies that LCs MVs can stimulate some processes such as the cell growth, angiogenesis induction, and the escape of blast cells from the detection by the immune system through the secretion of their components (4, 15). MVs secretion in leukemia is increased during the onset and progression of the disease and their ectopic secretion is associated with the increased invasion and progression towards the progressive stages (4). Moreover, these secretory components can result in multidrug resistance (MDR) in leukemias by transporting certain proteins (15). The significance of this issue is revealed when the plasma levels of some MVs are reduced following chemotherapy in leukemias, while an increase in their levels is observed sometime after the treatment (16).

It can be inferred that although these types of MVs are not resistant to treatments, their increase following the treatment may be considered a marker of the relapse phase during the disease. Hence, the assessment of these vesicles can provide a better understanding of chemotherapy-resistant leukemias. Given that the secretion of MVs components can have a significant impact on critical processes of LCs, it seems that their presence could play a key role in the progression and prognosis of leukemias (17, 18). In this review article, we attempt to examine the role of MVs expression in the progression of leukemia and their potential effects on the prognosis of these abnormalities. 

### Microvesicles in the progression of acute lymphocytic leukemia 

Acute lymphocytic leukemia (ALL) is the most common childhood leukemia, which results from the clonal proliferation of lymphoid precursors in BM (15). Most studies have indicated the miRs are as the most prevalent components in ALL MVs components. MiR-150 is among these miRs, in which its expression is decreased in ALL Nalm-6 cell line MVs (16, 17). MiR-150 can inhibit the differentiation of lymphocytes by preventing the cell transition from pro-B to the pre-B stage. On the other hand, miR-150 can directly contribute to the reduced expression of the c-Myc transcription factor which is involved in controlling the development of lymphocytes (17, 18). Thus, it seems that the reduced expression of miR-150 can, directly and indirectly, increase the upregulation of the immature lymphoid cells in ALL. 

Similar to miR-150, the expression levels of some other miRs such as miR-15b, miR-424, and miR-101 are decreased in B-ALL Nalm-6 cell line MVs. It has been shown that the reduced expression of these miRs can be associated with the increased expression of a number of genes such as cyclin D1 (CCND1) and B-Cell CLL/Lymphoma 9 (BCL9) in this cell line (16). BCL9 is a component of the Wnt/ß-catenin signalling pathway that plays an important role in the regulation of self-renewal, proliferation, and differentiation in normal and malignant cells. On the other hand, BCL9 can increase ß-catenin activity which has a key role in the increases self-renewal and maintenance of leukemic stem cells (LSCs) (19, 20). CCND1 is a cell cycle regulator with a recognized role in the control of the G1/S transition by regulating the function of cyclindependent kinases (CDKs) (21). Song et al. (21) have shown that CCND1 overexpression in T and B-cell lymphomas may function as an oncogene through the activation of the proliferation and differentiation processes. Similar to B-ALL Nalm-6 cell line, CCND1 and BCL9 overexpression as a post-translational phenomenon following the reduced expression of miR15b, miR-424, and miR-101 in MVs may be useful as a potential therapeutic target in ALL patients. 

**Fig.1:**
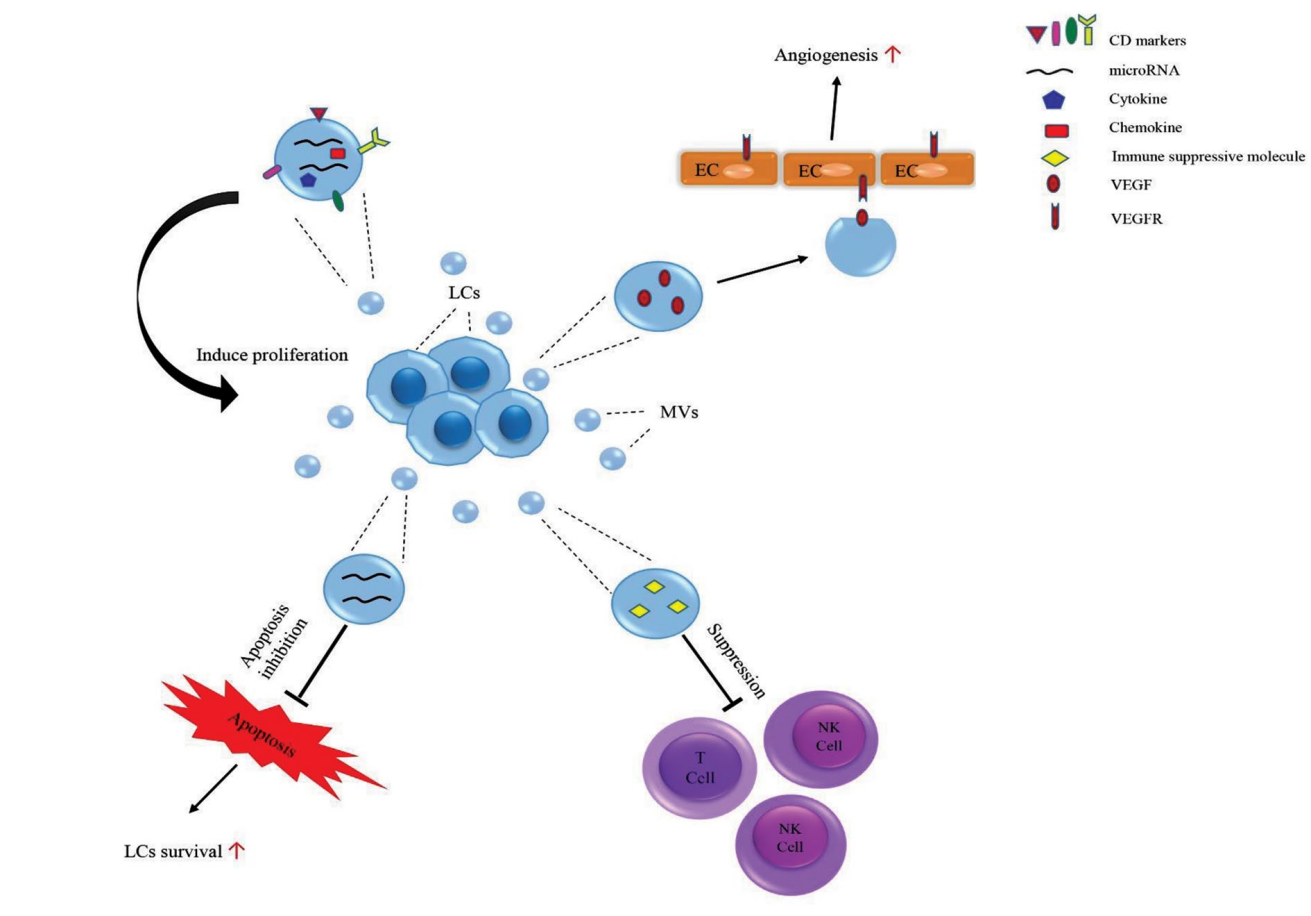
The mechanism of MVs in leukemia progression. LCs-derived MVs affect the cell fate via direct binding to receptors of the target cells. In fact, these vesicles can play a role in vital processes of target cells by transfer different agents such as microRNAs, cytokines, and chemokines. LCs-derived MVs as autocrine positive feedback could be a cause of LCs progression. Also, MVs can increase LCs survival by a decrease in anti- leukemia activity via the suppression of the immune cells, the reduction of proapoptotic proteins, and the induction of angiogenesis. Therefore, LCs-derived MVs play an important role in the progression of leukemia via disrupting the balance of these processes. MVs; Microvesicles, LCs; Leukemic cells, ECs; Endothelial cells, VEGF; Vascular endothelial growth factor, and VEGFR; VEGF receptor.

Unlike the Nalm-6 cell line in which the reduced expression of some MVs miRs have been shown, the accumulation of miR17-92 cluster containing (e.g. miR-92a, miR-92b, miR-18a, miR-18b, and miR96) in T-ALL Jurkat cell line MVs has been reported by a study of Li et al. (16). Many tumor suppressor proteins such as phosphatase and Tensin homolog (PTEN), and apoptotic proteins including BIM (BCL2) and E2F transcription factor1 (E2F1) can be targeted by this aberrantly expressed miRs in this cell line (16, 22). BIM induces apoptosis via activating a number of proapoptotic family members such as BCL-2 associated proapoptotic x protein (Bax) and BCL-2 antagonist killer-1 (Bak) (23). On the other hand, PTEN can phosphorylate and activate BCL2associated agonist of cell death (BAD), which leads to the induction of apoptosis (24). E2F1 is a protein that plays an important role in the regulation of apoptosis mediators such as retinoblastoma protein (pRb) and murine-double-minute-2 (MDM-2). PRb is a negative cell cycle regulator that can be activated by binding E2F1 in a hypo-phosphorylated form. On the other hand, MDM-2 activates P53 and controls the cell cycle during the transition from G1 to S phase (25). Also, it has been shown that miR-1246-containing MVs can suppress the function of P53 and prevent the LCs apoptosis (Fig .1) (16).

Similar to T-ALL Jurkat cell line, the reduced expression of these proapoptotic proteins due to the secretion of the above-mentioned miRs from ALL MVs results in LCs survival. Since miR-containing MVs can play a critical role in the progression of ALL by inhibition of apoptosis, as well as increasing the survival and proliferation of LCs, the overexpression of this miRs can be a poor prognostic marker in ALL.

### Chronic lymphocytic leukemia-derived microvesicles

Chronic lymphocytic leukemia (CLL) is a leukemia of apoptosis-resistant mature B-cells which is characterized by the expression of CD5^+^, CD19^+^, CD23^+^, CD10^+^, CD20+, CD22^+^, CD79α, and CD79ß. These cells can clonally aggregate in PB, BM, lymph nodes, and spleen (26). Some MVs are derived from B-cell CLL possessing AXL receptor tyrosine kinase (27). AXL acts as an active regulator of kinases such as Lyn and phosphatidylinositol 3-kinase (PI3K)/AKT serine-threonine kinase (4). On the other hand, AXL-containing MVs can enhance the expression of vascular endothelial growth factor (VEGF) by activating the AKT/mammalian target of rapamycin (mTOR)/P70S6K/hypoxia-inducible factor-1a (HIF-1a) signalling pathway in BM stromal cells (BMSCs) (28). VEGF has a well-known role in inducing angiogenesis by binding to its cognate receptor on the endothelial cells (Fig .1) (4, 28). On the other hand, these MVs can promote the expression of CCND1 and c-MYC by activating the AKT/ß-catenin signalling pathway in BMSCs (29). CCND1 and c-MYC play important roles in cell cycle regulation. Several studies have shown that the induction of the expression of CCND1 and c-MYC by AXL- containing MVs can dysregulate the cell cycle in BMSCs and lead to the increased proliferation of these cells (4, 27, 28, 30). Therefore, this is inferred that the release of AXL from B-cell CLL MVs may result in a higher BM density due to angiogenesis and BMSCs proliferation induction. In these conditions, high BM density can cause a problem in BM aspiration in CLL patients.

Another type of MVs in B-CLL patients has the certain CD markers on their surface that can indicate their origin. The overexpression of CD20, CD19, CD37, and CD52 is among the changes observed on MVs surface in CLLpatients (Table 1) (7, 28, 31, 32). Interestingly, De Luca et al. (31) in their study on newly diagnosed B-CLL patients showed that an increase in the number of MVs bearing these CD19 and CD37 had a direct correlation with a high tumor burden and an inverse relationship with the overall survival. This finding suggests that the overexpression of MVs bearing these CD markers could be a poor prognostic biomarker for the patient survival. CD52 is a specific target of humanized monoclonal antibody Alemtuzumab (CAMPATH-1H) used for the treatment of relapsed or refractory CLL (33). A higher expression of CD52 in serum vesicles has been reported in a B-CLL patient with poor risk karyotype (17p- and 11q) and more advanced disease (Rai stage III) (7). In addition, Albitar et al. (34) demonstrated a high level of soluble CD52 in the plasma of patients which is inversely associated with the plasma concentration of Alemtuzumab and also can cause a nearly 4-fold increase in the risk of death in CLL patients. Therefore, the presence of MVs bearing CD52 could be a poor prognostic biomarker for the CLL progression toward the advanced stage. Also, the assessment of MVs bearing CD52 in CLL patients can provide useful information for the analysis of Alemtuzumab therapy and minimal residual disease (MRD).

MiRs are the components of CLL MVs that can affect various aspects of LCs function by binding their targets. Yeh et al. (35) have shown that MVs-containing miR-150 and miR-155 can boost B-cell receptor (BCR) expression in B-cells via the secretion of their components. Also, they showed the increased BCR activation through a-IgM stimulation which can lead to the increased secretion of MVs containing these miRs. BCR is the main functional receptor on B-cells, and several processes of these lymphocytes, including antibody production, are linked to its downstream signalling pathways (36). Therefore, a change in the expression of BCR can affect the activity of B lymphocytes. Since BCR signalling plays an essential role in the proliferation and maintenance of malignant B-cells, increasing the activity of the cell signalling via the secretion of miR-150 and miR-155 from MVs may be associated with the survival of LCs in CLL (35, 37). Considering the cross-talk between MVs containing biological molecules and CLL cells, the assessment of the impact of MVs on the processes of LCs such as maintenance, proliferation as well as the response therapy and MRD in this disease can reveal the prognostic value of MVs expression in the prediction of CLL progression.

### Circulating microvesicles in acute myeloid leukemia

Acute myeloid leukemia (AML) is a hematological malignancy associated with a rapid proliferation of myeloblasts in BM and their release into PB (38). Studies have shown that MVs secreted from LCs, especially in AML patients, can induce the proliferation, migration, and apoptosis inhibition in these patients (39). Additionally, these MVs can suppress the immune system through the release of immune suppressive molecules such as transforming growth factor beta1 (TGFß1), Fas ligand, programmed cell death 1 ligand (PD-L1), CD39, CD73, MHC class I polypeptide-related sequence A (MICA), and MHC class I polypeptide-related sequence B (MICB) (11, 40). Therefore, these vesicles as the immune suppressors are able to decrease anti-leukemia activity and play a role in LCs escape from the immune defense processes. Natural killer cells (NK cells) play a vital role in the eradication of tumor cells in a wide range of cancers, including leukemia (41). The function of these cells is controlled by activating and inhibiting the receptors expressed on their surface. NKG2D is among the active receptors located on the surface of NK cells that its expression is a sign of the active function of NK cells (42). In a study conducted on the function of these types of cells in AML patients, Szczepanski: et al. (43) found that the expression of NKG2D on NK cells is decreased following the secretion of TGFß1 from blast-derived MVs. In fact, this type of MVs suppresses the function of NK cells by the secretion of their components (Fig .1). On one hand, interleukin-15 (IL-15) can protect NK cells from the adverse effects of these MVs. Considering the role of NK cells in the killing of LCs, the suppression of NKs function by TGFß1-containing MVs seems to provide conditions for LCs survival and thereby AML progression. Therefore, an increase in this type of MVs can be a poor prognostic factor in AML. 

**Table 1:** Prognostic value of CD markers’ expression in leukemia MV


Leukemia	CD markers	Cho.	Expression	Prognosis	Ref.

CLL	CD19	16p11.2	High	Can be associated with CLL progression via increased BCR signalling in B-cells	(31, 32, 44)
	CD37	19q13.33	High	Associated with the progression of pre-B to mature B-cell lymphocyte and subsequently increased proliferation	(31, 45)
	CD20	11q12.2	High	Can be associated with CLL progression	(28, 32, 46)
	CD52	1p36.11	High	Maybe associated with poor prognosis via increased progression and invasion of B-cells	(7, 28, 33)
AML	CD13	15q26.1	High	Poor prognosis via increased migration of cells	(47, 48)
	CD33	19q13.41	High	Associated with increased myeloid blast cells	(40, 47, 49)
	CD117	4q12	High	Can be associated with poor prognosis via interaction with SCF and subsequently increased blast cells survival	(40, 47, 50)
	CD34	1q32.2	High	Maybe associated with increased blast cells	(40, 47, 51)
CML	CD34	1q32.2	High	Can be associated with increased blast cells	(12)
	CD123	Xp22.33	High	Can be associated with poor prognosis by increased proliferation	(12)


CD; Cluster of differentiation, MVs; Microvesicles, CLL; Chronic lymphocytic leukemia, AML: Acute myeloid leukemia; CML; Chronic myeloidleukemia, BCR; B-cell receptor, and SCF; Stem cell factor.

On the other hand, it may be inducing the expression of IL-15 by new immunotherapy agents which can protect NK cells from the adverse effects of these MVs. The relapse is a problem can complicate the process of the treatment for AML patients. Studies displayed that in addition of the genetic background of individuals, some AML MVs contain proteins that play a crucial role in drug resistance and relapse in this disease (52). AML MVs contain chemoattractants such as I-309, monocyte chemotactic protein 1 (MCP-1), and MCP4, which can lead to the resistance of AML blasts to chemotherapy by trafficking, proliferation, migration, and mobilization of these blasts (52, 53). Considering the impact of I-309, MCP-1, and MCP-4 MVs on AML blasts drug resistance, it may be targeting these MVs by chemotherapeutic agents reduce the MRD, as well as relapse in this disease. Another AML MVs also have procoagulants such as TF which is a component of AML MVs that could be associated with hypercoagulable state and increased the risk of thrombosis in this malignancy (52). Considering some AML MVs contain VEFG, the secretion of them can lead to elevated angiogenesis and hence increased the chance of thrombosis (4). It can be mentioned that the disturb balance between pro- and anti-coagulant factors by AML MVs may have a potential role in the incidence of thrombotic events in AML patients. 

The increased expression of myeloid markers is an early indicator of the presence of blasts in BM and PB of AML patients. Some of these blasts can secrete MVs bearing myeloid-specific CD markers, which can be thus distinguished from MVs of normal cells. Several studies indicated that the expressions of CD13, CD34, CD117, and CD33 in blast-derived MVs in AML patients can be associated with the presence of activated blasts in this disease (Table 1) (11, 40, 47). Regarding CD13, CD34, CD117, and CD33 are the immature myeloid specific markers; it seems that a higher expression of these markers in AML MVs may be displayed the presence of active myeloid neoplastic clone in BM. However, few studies indicated the possible correlation between the AML MVs markers and clinical findings or response/resistance to the therapeutic agents of this disease. Therefore, more studies are required to reveal the prognostic value of AML MVs markers in clinical outcomes and disease aggressiveness.

MiRs are another type of secreted MVs in AML patients. Among these miRs, the expression of miR-155 has been shown to increase in AML MVs. Interestingly, the presence of this type of MVs is associated with the increased white blood cell (WBC) counts and a complex karyotype such as genotype FLT3-ITD in combination with NPMc+ (37, 54). Since miR-155-containing MVs are associated with high WBC counts and the FLT3-ITD combination, it seems that a higher level of this MVs has a poor prognostic value in AML patients. Unlike miR155, the increase in some miRs (like miR34a) can be associated with a favorable prognosis. Wang et al. (38) in their recent study demonstrated that the increased miR34a level in MVs of KG1a cell line can be associated with the suppression of proliferation and induce apoptosis in this cell line. In this situation, miR-34a could act as a tumor suppressor by affecting factors involved in apoptosis such as caspase-3 and T cell immunoglobulin mucin-3 (Tim-3). According to these findings, it may be inducing the expression of miR34a level in KG1a cell line MVs and transferring them to patients with AML as new therapeutic agents which can improve the management of these patients. Since AML MVs can be associated with AML progression, we believe that the MVs may be used as an independent prognostic biomarker in monitor AML progression. However, further studies are required to substantiate this notion.

### Chronic myeloid leukemia microvesicles

Chronic myeloid leukemia (CML) is a clonal myeloproliferative disorder characterized by the presence of translocation (9, 22) and a range of immature myeloid cells (12, 13). Several recent study demonstrated that MVs which are derived from LAMA84 CML cell line through secretion of interleukin-8 (IL-8) can induce the intercellular adhesion molecule 1 (ICAM-1) and vascular cell adhesion molecule 1 (VCAM-1) expression in human umbilical vein endothelial cells (HUVECs), which is associated with an increase in the adhesion and migration of CML cells (4, 11, 55, 56). Considering the fact that ICAM-1 and VCAM-1 can mediate CML cells adhesion to endothelial cells, it may be similar to LAMA84 CML cell line, the release of IL-8 from MVs-derived CML can induce thrombotic process in this malignancy.

MiR-containing MVs are secreted by LSCs in CML, which can reflect the abnormal function of these stem cells. Chen et al. (13) in a recent study have shown that the overexpression of miR 23-27-24 cluster and onco-miR cluster, which includes several miRs such as miR-17, miR19a, miR-19b, miR-20a, and miR-92a that play an important role in development and proliferation of CML K562 cell line. The increase in miR 23-2724 cluster can enhance angiogenesis by promoting angiogenic signalling including Ras/MAP kinase and vascular endothelial growth factor receptor 2 (VEGFR2) signalling in endothelial cells (56, 57). On the other hand, Tadokoro et al. (58) exhibited that the co-culture of the K562 cell line containing miR-210 in hypoxic conditions with HUVECs enables miR-210 to induce angiogenesis by reducing ephrin A3 (EFNA3) as a negative regulator of angiogenesis. Although a high level of immature myeloid progenitors is the main cause of the thrombotic event in CML, it may be similar to K562 cell line, the release of miRs-210 from CML MVs in hypoxic conditions induces the thrombotic activity in CML patients.

It has been demonstrated several miRs derived from K562 cell line MVs including miR-27b, miR-24, miR-23b, miR126, has-let-7f, has-let-7a, miR-1249, miR-185, miR-7, and miR-130blet-7b may contribute to the development of this cell line. These miRs have been demonstrated to be involved in a number of biological processes, including development, differentiation, apoptosis, and proliferation of K562 cell line. For example, miR-7 may play a role in leukemogenesis by abnormally regulating their target genes such as retinoblastoma 1 (RB1), breakpoint cluster region (BCR), phosphatidylinositol4,5- bisphosphate 3-kinase catalytic subunit delta (PIK3CD), phosphoinositide-3-kinase regulatory subunit 3 (PIK3R3), BCL2 like 1 (BCL2 L1), and v-raf-leukemia viral oncogene 1 (RAF1). Furthermore, miR-126 can activate the PI3K/AKT signalling pathway by affecting the v-crk avian sarcoma virus CT10 oncogene homolog (CRK) (13).

A large number of these target genes were involved in the activation of PI3K/AKT signalling pathway, cell cycle, and P53 signalling, which involved in various processes such as development, proliferation, and apoptosis of LCs (13, 59). Since most of these pathways are involved in the vital processes of LCs; therefore, miRs derived MVs may contribute to the uncontrolled development, as well as resistance to apoptosis in LCs. Furthermore, Zhang et al. (60) have shown that a high level of miR-146b-5p in K562 cell line MVs can promote hematopoietic cells to a leukemic state. Silencing NUMB gene in the recipient cells by miR-146b-5p in K562-MVs is a possible mechanism for leukemic transformation of hematopoietic cells. NUMB can inhibit LCs proliferation by preventing degradation of p53 and Notch signalling pathway, which is involved in apoptosis and cell cycle respectively (Table 2) (61). So that, it is implied that MVs containing miR-146b-5p can have a role in the promotion of LCs proliferation by silencing NUMB gene indirectly.

Certain MVs are secreted in CML patients bearing CD markers which are indicating their derivation from blast cells. For example, the overexpression of CD34 and CD123 in MVs derived from CML CD34+ blasts can be associated with an increase in immature cells in PB (12). The CD123 or interleukin 3 receptor subunit alpha (IL-3RA) is one of the markers expressed on the majority of CD34+/CD38-MVs derived from CML blasts (62, 63). IL-3 is a pleiotropic cytokine that functions as a multi-colony stimulating factor (multi-CSF), and its binding to its receptor (CD123) on hematopoietic cells can be associated with their development in BM (Table 1) (63). Given the low expression of CD123 in normal hematopoietic cells, the overexpression of this marker on MVs derived from CML cells indicated the stimulation of CML cells proliferation. Due to constitutive role of MVs in biological process and clinical finding in CML, MVs could be a powerful prognostic biomarker and target therapy in this malignancy.

**Table 2:** Prognostic value of miRs contents of MVs in leukemia


Leukemia	miRs	Cho.	Expression	Prognosis	Ref.

ALL	miR-150	19q13.33	Low	Good prognosis via transition of B-cell from pro-B to pre-B and subsequent increase in differentiation and development of B-cells	(16, 18)
	miR-101	1p31.3	Low	Associated with a poor prognosis via decreased apoptosis	(15, 16)
	miR-424	Xq26.3	Low	Associated with poor prognosis via induction of cell-cycle and subsequent increase of proliferation	(16, 64)
	miR-15b	3q25.33	Low	Can be associated with poor prognosis via decrease of caspase signalling cascade and decreased apoptosis	(16, 65)
	miR-1246	2q31.1	High	Poor prognosis via down-regulation of P53 and subsequently decreased apoptosis	(16)
CLL	miR-155	21q21.3	High	Can be associated with a poor prognosis via activating BCR signalling and increased proliferation	(35, 37, 64)
	miR-150	19q13.33	High	Maybe associated with a poor prognosis via inhibiting lymphocyte differentiation and decreased B-cell maturation	(18, 35)
AML	miR-34a	1p36.22	High	Good prognosis via the induction of apoptosis and decreased proliferation	(38, 66)
	miR-155	21q21.3	High	Associated with a poor prognosis via increased proliferation	(37, 39, 64)
CML	miR-210	11p15.5	High	Can be associated with poor prognosis through induced angiogenesis and cell-cycle	(11, 40)
	miR-146b-5p	10q24.32	High	Poor prognosis via inhibition of NUMB, Notch 2, BRCA1 and subsequently increased proliferation	(60)


MVs; Microvesicles, ALL; Acute lymphocytic leukemia, CLL; Chronic lymphocytic leukemia, AML; Acute myeloid leukemia, CML; Chronic myeloid leukemia, BCR; B-cell receptor, Cho; Chromosome, NUMB; Endocytic adaptor protein, and BRCA1; DNA repair associated.

MVs are the smallest subclass of vesicles secreted by the external budding from the membrane of cells in physiologic and pathologic states (1-4). These vesicles contain various biological agents and can release their components via interaction with target cells, leading to functional and phenotypic changes in these cells (3, 8, 9). MVs can be secreted from LCs in hematological malignancies and play an important role as bioactive vesicles. In addition, these particles can carry different biological mediators such as miRs, cytokines, and chemokines (4). The secretion of these agents, especially miRs, from MVs can cause genetic changes in target cell due to their effects on the regulation of the gene expression. For example, miRs-containing MVs can be associated with the ALL progression and prognosis by the impact on molecules and signalling pathways that involved in a vital process of LCs such as differentiation, proliferation, and apoptosis. On the other hand, LCs derived MVs can be recognizable via the expression of specific CD markers showing their origin. Elevated levels of some MVs CD markers expression such as CD52 can be associated with the CLL progression toward advanced stage (33). Similarly, a higher expression of myeloid progenitor CD markers in AML MVs may display the presence of the active myeloid neoplastic clone in BM (11, 40, 47). Hence, flow cytometry analysis of MVs related to CD markers in alongside the specific diagnostic CD markers in leukemias can reveal their prognostic value in disease progression. In addition to the biological process of LCs, the secretion of MVs components such as ICAM-1, VCAM-1, TF, and VEGF, which mediate the thrombotic event and angiogenesis process, can be associated with the incidence of unfavorable clinical outcomes in leukemia patients. Furthermore, MVs containing TGF-ß can act as anti-leukemic agents by suppression of the immune cell activation in AML (43). It seems that these MVs function may have a role in drug resistance, as well as AML relapse by helping LCs to escape from the defective immune system. Accordingly, MVs can be used as prognostic biomarkers for the disease monitoring in hematological malignancies. It is conceivable that the inactivation of MVs function by new drug strategies can minimize the side effects of these particles on the clinical finding of leukemia patients and leads to improve the condition of those patients.

## Conclusion

Leukemia-derived MVs can play an indispensable role in LCs maintenance by the impact on the vital process including survival, proliferation, and apoptosis of these cells. So that, MVs can have a crucial role in the leukemias progression. In spite of the important role of MVs in LCs survival, most studied of MVs function has been done on leukemic cell lines. Therefore, further clinical trials for a better understanding of MVs mechanisms in leukemias are required to confirm the relationship between these particles with the leukemias prognosis. It may reduce their undesirable effects by preventing the secretion of MVs components from leukemic cells, removing them from the circulation, and blocking the binding of MVs to their corresponding receptors by a new therapeutic approach that leads to improving the patients’ condition.
